# The Mini Mental State Examination: Review of cutoff points adjusted for schooling in a large Southern Brazilian sample

**DOI:** 10.1590/S1980-57642010DN40100006

**Published:** 2010

**Authors:** Renata Kochhann, Juliana Santos Varela, Carolina Saraiva de Macedo Lisboa, Márcia Lorena Fagundes Chaves

**Affiliations:** 1 Medical Sciences Post-Graduate Course, UFRGS School of Medicine.; 2 Dementia Clinic, Neurology Service, Hospital de Clínicas de Porto Alegre.; 3 Internal Psychology Department, UNISINOS School of Psychology.; 4 Internal Medicine Department, UFRGS School of Medicine.

**Keywords:** Mini Mental State Examination, cognition, cognitive assessment, educational attainment, cutoffs

## Abstract

The increase in life expectancy can influence the prevalence of dementias in the population. Instruments that evaluate cognitive functions such as the Mini Mental State Examination (MMSE) are necessary for the investigation of dementia. The supposition that patient score on the MMSE can be influenced by academic level points to the need for establishing cut-off values that take into account educational level. The aim of this study was to review MMSE cut-off values adjusted for schooling in a large southern Brazilian sample.

**Method:**

Demographic data and MMSE scores of 968 subjects, of which 162 were dementia patients and 806 healthy participants, were analyzed. The sample was grouped according to education. The cut-off values were established by ROC Curve analysis.

**Results:**

The total sample mean age was 70.6±7.3 years, and the mean years of education was 7.2±5.3. The cut-off score of 23 points (sensitivity=86%, specificity=83%) was observed as the optimal level to detect dementia on the MMSE instrument for the overall sample. Regarding level of schooling, the cut-off values were: 21 for the illiterate group (sensitivity=93%, specificity=82%), 22 for the low education group (sensitivity=87%, specificity=82%), 23 for the middle education group (sensitivity=86%, specificity=87%) and 24 for the high education group (sensitivity=81%, specificity=87%).

**Conclusions:**

The cut-off values revealed by this analysis, and adjusted for level of schooling, can improve the clinical evaluation of cognitive deficits.

## Abstract (pt)

A elevação da expectativa de vida pode influenciar na prevalência das demências na população. Instrumentos que avaliem as funções cognitivas, como o Mini Exame do Estado Mental (MEEM), são necessários para a investigação de demência. A suposição de que o resultado do MEEM de um paciente pode ser influenciado pelo nível de escolaridade demonstra a necessidade do estabelecimento de pontos de corte que levem em consideração a escolaridade. O objetivo deste estudo foi revisar os pontos de corte do MEEM ajustados para a escolaridade em uma grande amostra do sul do Brasil.

**Método:**

Dados demográficos e escores do MEEM de 968 indivíduos, 162 pacientes com demência e 806 participantes saudáveis foram analisados. A amostra foi agrupada de acordo com a educação. Para estabelecer os pontos de corte foi utilizada a Curva ROC.

**Resultados:**

A média de idade da amostra total foi 70,6±7,3 e a média de anos de estudo foi 7,2±5,3. O ponto de corte 23 (sensibilidade=86%), (especificidade=83%) foi o ponto que melhor detectou demência na amostra total. Considerando o nível de escolaridade, os pontos de corte foram: 21 no grupo de analfabetos (sensibilidade=93%, especificidade=82%), 22 no grupo de baixa escolaridade (sensibilidade=87%, especificidade=82%), 23 no grupo de média escolaridade (sensibilidade=86%, especificidade=87%) e 24 no grupo de alta escolaridade (sensibilidade=81%, especificidade=87%).

**Conclusões:**

Os pontos de corte, quando se considera a escolaridade, podem aperfeiçoar a avaliação clínica dos déficits cognitivos.

The significant increase in the elderly population in recent years because of greater life expectancy, has led to an increase in the prevalence of dementias.^1^ These diseases occur mainly during the aging process and increase exponentially as a function of age. The dementias affect around 5% of the population above 65 years of age and, among individuals aged 80 years, this frequency may reach 20% to 25% of the population.^2^ The dementias are syndromes characterized by a decline in cognitive functions that leads to a significant impairment in the activities of daily living, representing a decline compared to previous superior level of functioning.^3^

Instruments that evaluate cognitive functions, such as the Mini Mental State Examination (MMSE) are necessary for the corroboration of cognitive deficits.^4-6^ The MMSE is a screening test that should be used in individuals with suspected cognitive deficit, but it cannot be used to diagnose dementia. Dementia diagnosis should be made based on classification systems such as the International Statistical Classification of Diseases and Related Health Problems (ICD-10) and the Diagnostic and Statistical Manual of Mental Disorders (DSM-IV).

Since it was developed, the MMSE has been studied with different focus of interest. There are studies reporting research data where the MMSE is a component of a battery of tests to detect dementia cases.^7-10^ Other studies have focused on the performance of the MMSE in the general population.^11-15^

In 1982, Anthony and coworkers^16^ showed the results of sensitivity (87%) and specificity (82%) for the cutoff (23 case/24 non case) on the MMSE to detect dementia, and the cutoff they proposed is still used worldwide.^17-21^

Tombaugh and coworkers (1992)^22^ published a review of the MMSE. The authors showed that a higher level of Cronbach's Alfa coefficent (0.96) was obtained in a mixed group of patients, while the intermediate values of 0.68 and 0.77 were found in samples drawn from the community. The review concluded that the MMSE could be used to evaluate severity quantitatively of a cognitive deficit as well as changes over time, as was suggested in its development, but could not be used as a single tool for diagnosing dementia.

Fratiglioni and coworkers (1993)^23^ presented data showing that people with lower levels of education had lower scores on the MMSE, justifying the use of different cutoffs, adjusted for schooling years. By applying this method, these authors achieved no diagnostic mistakes in the dementia cases. Applying this notion, other studies have taken into account educational level or age to produce different cutoffs for the detection of dementia. However, there are no standard cutoffs for the MMSE with regards to education, since each study suggested different cutoffs.^20,24-28^

In Brazil, because the number of individuals with lower levels of education is large, adjusting cutoffs to schooling is very important to decrease false positives.^3,20.21,26.28,29^ The present study aimed to review cutoffs adjusted for educational level in a large southern Brazilian sample composed of healthy participants and dementia patients.

## Methods

### Sample selection

A cross-sectional study was conducted in a sample of 162 dementia patients and 806 healthy participants. The dementia patients were recruited from the Dementia Clinic, Neurology Service, Hospital de Clínicas de Porto Alegre, and fulfilled the DSM-IV criteria for dementia, Alzheimer’s disease and vascular dementia.^3^ Healthy participants were randomly selected from different sectors of the same hospital (relatives, caregivers and visitors). The inclusion criteria for healthy participants were to be functionally independent and cognitively healthy on the Clinical Dementia Rating scale (CDR=0).^30^ The exclusion criteria were presence of any psychiatric or neurological disease and use of psychoactive drugs. All participants underwent evaluation with the Mini Mental State Examination (MMSE), Brazilian version.^26^

The total sample comprised 633 women (65%), had a mean age of 70.6±7.3 years (range: 60-92) and mean years of education of 7.2±5.3(range: 0-35). Forty percent of dementia patients had mild dementia (CDR=1), 43% moderate dementia (CDR=2) and 17% severe dementia.

To review the cutoffs adjusted for schooling, the participants were subdivided into 4 groups: illiterate, lower educational level (1-5 years), middle educational level (6-11 years) and higher educational level (≥12 years). These criteria have been analyzed in a previous study,^31^ but for this study illiterates formed a separate group.

The groups were composed of 15 dementia patients and 57 healthy participants (illiterate group), 77 dementia patients and 338 healthy participants (lower educational level group), 43 dementia patients and 234 healthy participants (middle educational level group) and 27 dementia patients and 177 healthy participants (higher educational level group).

### Mental state evaluation

The Mini Mental State Examination is a tool for cognitive screening used worldwide for global evaluation.^6,7.24,32-34^. It was developed by Folstein et al. in 197535 and has versions in different languages and countries.^7-9.11-13.17-19.24,25.34,36^ There are also versions validated for the Brazilian population.^26,29.37^

This instrument was developed as a brief cognitive evaluation (5-10 min) in psychiatric patients. The test was named "mini" because it focuses only on the cognitive aspects of mental functions and excludes questions about mood, abnormal mental phenomena and thought patterns.^35^

The MMSE evaluates several cognitive domains: temporal and spatial orientation, working and immediate memory, attention and calculus, naming of objects, repetition of a sentence, execution of commands, comprehension and writing task execution, comprehension and verbal task execution, planning and praxis.

In all items, each correct answer scores one point and each incorrect answer scores zero. The maximum score that can be obtained is thirty and the minimum is zero. The lower the score, the more significant is the impairment.

The MMSE was recommended as a screening tool for global cognitive testing by the Brazilian Academy of Neurology^38^ and by the American Academy of Neurology.^39^

### Statistical analysis

Descriptive statistics (mean, SD, and relative frequency) were calculated for demographic data and MMSE. Student’s t test was used for comparison of parametric data, and the Chi-square test for categorical data. ROC curves (Receiver-Operating Characteristic Curves) were constructed to establish the cutoff points.

The statistical analysis was carried out with the *Statistical Package for the Social Sciences* for Windows version 13.0 (SPSS Inc., Chicago, IL, USA.). Sensitivity, specificity, positive and negative predictive values, and their 95% confidence interval levels for cutoff points were calculated using the Epicalc package from R project for Statistical Computing 2.8.1(R Foundation, Auckland, New Zealand).

The study was approved by the Ethics Committee for Medical Research of Hospital de Clínicas de Porto Alegre. All subjects signed an informed consent before being enrolled in the study.

## Results

The majority of the demographic variables were significantly different between the dementia patients and the healthy participants group. The age of dementia patients and healthy participants were not significantly different in the lower education, middle education and higher education groups, Also, in the illiterate and higher education groups, the sex of the dementia patients and the healthy participants were also not significantly different. Demographic data of the sample is presented in Table 1.

**Table 1:** Demographic data on dementia patients and healthy elderly participants in the total sample by educational level.

Sample	Demographic variables	Dementia patients	Healthy participants	P value
Total	Age* (mean (SD))	72.1 (7.7)	70.3 (7.2)	0.005
	Sex** Female (N (%))	84 (52)	549 (68)	<001
	Educational level* (mean (SD))	6.1 (4.3)	7.4 (5.4)	0.002
	MMSE* (mean (SD))	16.7 (6.7)	26.3 (3.0)	<001
Illiterate	Age* (mean (SD))	76.5 (7.3)	66.7 (6.3)	<001
	Sex** Female (N (%))	11 (73)	50 (88)	0.168
	MMSE* (mean (SD))	15.0 (5.7)	25.2 (3.3)	<001
Lower education	Age* (mean (SD))	71.1 (6.7)	70.3 (7.1)	0.353
	Sex** Female (N (%))	37 (48)	240 (71)	<001
	MMSE* (mean (SD))	16.4 (6.4)	25.4 (3.1)	<001
Middle education	Age* (mean (SD))	71.6 (8.7)	70.8 (7.1)	0.564
	Sex** Female (N (%))	23 (53)	171 (73)	0.010
	MMSE* (mean (SD))	16.6 (7.1)	27.1 (2.6)	<001
Higher education	Age* (mean (SD))	73.2 (7.5)	70.9 (7.5)	0.155
	Sex** Female (N (%))	13 (48)	88 (49)	0.879
	MMSE* (mean (SD))	18.6 (7.1)	27.4 (2.7)	<001

* Student's t test;

** Chi-square test.

**Figure 1:**
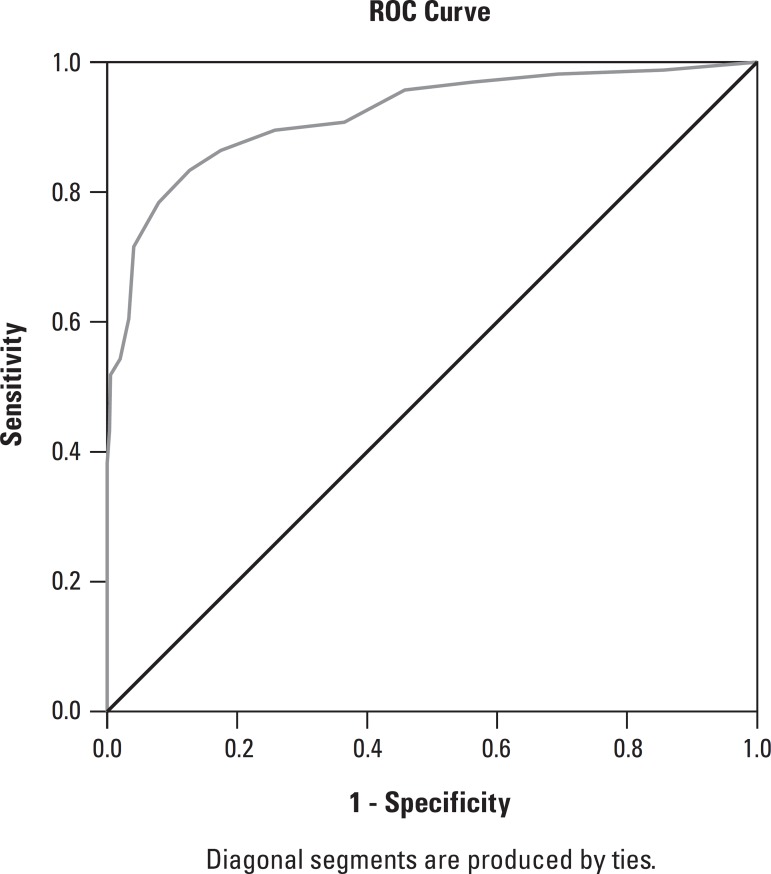
ROC curve of total sample.

According to the ROC curve analysis, the Brazilian-Portuguese MMSE version presented high diagnostic accuracy for identifying dementia in this sample (AUC=0.92, 95% CI=0.89-0.94) (Figure 1). The optimal cutoffs were determined by finding the values that allowed the best balance between sensitivity and specificity. For the majority of the cutoffs, the sensitivity was higher than the specificity because MMSE is a mental state screening instrument.

A range of possible cutoff values is shown in Table 2. The cutoff of 23 in the total sample yielded a sensitivity of 86%, specificity of 83%, a 50% positive predictive value and 97% negative predictive value. In the illiterate group, the cutoff of 21 yielded sensitivity of 93%, specificity of 82%, a 58% positive predictive value and 98% negative predictive value. In the lower education group, the cutoff of 22 yielded sensitivity of 87%, specificity of 82%, a 53% positive predictive value and 96% negative predictive value. For the middle education group, the cutoff of 23 showed sensitivity of 86%, specificity of 87%, a 55% positive predictive value and 97% negative predictive value. Finally, for the higher education group, the cutoff of 24 showed sensitivity of 81%, specificity of 87%, a 50% positive predictive value and 97% negative predictive value.

**Table 2:** Cut-off score on the Mini Mental State Examination obtained from coordinates of the ROC curve, and corresponding sensitivity, specificity, Positive Predictive Value (PPV) and Negative Predictive Value (NPV).

Sample	Cutoffs	Area under the curve	Sensitivity(CI 95%)	Specificity(CI 95%)	PPV(CI 95%)	NPV(CI 95%)
Total	20	0.918 (0.892-0.945)	72 (64-78)	96 (94-97)	78 (70-84)	94 (93-96)
	21		78 (72-84)	92 (90-94)	66 (60-73)	95 (94-97)
	22		83 (77-88)	85 (83-87)	53 (47-59)	96 (95-97)
	23		**86 (81-91)**	**83 (79-85)**	**50 (44-55)**	**97 (95-98)**
	24		89 (84-94)	74 (71-77)	41 (36-46)	97 (96-98)
	25		91 (86-94)	63 (60-67)	33 (29-38)	97 (95-98)
Illiterate	20	0.955 (0.904-1.000)	87 (65-97)	93 (84-98)	76 (54-92)	96 (89-99)
	21		93 (73-99)	82 (41-91)	58 (39-76)	98 (91-99)
	22		**93 (73-99)**	**74 (61-84)**	**48 (31-66)**	**98 (89-99)**
	23		93 (73-99)	68 (56-79)	44 (28-60)	97 (89-99)
	24		100 (83-100)	63 (50-75)	42 (27-58)	100 (92-100)
	25		100 (83-100)	49 (36-62)	34 (21-48)	100 (90-100)
Lower education	20	0.911 (0.868-0.953)	75 (65-84)	93 (90-96)	72 (62-81)	94 (91-96)
	21		82 (72-89)	88 (85-92)	62 (53-71)	95 (93-97)
	22		**87 (78-93)**	**82 (78-86)**	**53 (44-62)**	**96 (94-98)**
	23		89 (81-95)	76 (72-81)	47 (39-55)	97 (94-99)
	24		92 (85-97)	64 (57-67)	36 (29-43)	97 (94-99)
	25		92 (85-97)	49 (44-55)	29 (24-35)	96 (93-99)
Middle education	20	0.934 (0.886-0.982)	74 (60-86)	99 (97-99)	94 (83-99)	95 (92-98)
	21		77 (63-87)	96 (93-98)	78 (65-89)	96 (93-98)
	22		84 (71-92)	93 (89-96)	68 (55-79)	97 (94-99)
	23		**86 (74-94)**	**87 (83-91)**	**55 (43-66)**	**97 (94-98)**
	24		86 (74-94)	84 (78-98)	49 (38-60)	97 (94-98)
	25		91 (79-97)	77 (71-82)	42 (32-52)	98 (95-99)
Higher education	20	0.907 (0.836-0.977)	48 (30-66)	97 (94-99)	72 (50-88)	92 (88-96)
	21		63 (45-79)	96 (92-98)	71 (52-86)	94 (90-97)
	22		67 (48-82)	94 (89-97)	62 (44-78)	95 (91-97)
	23		74 (56-87)	92 (87-95)	58 (42-74)	96 (92-98)
	24		**81 (65-93)**	**87 (82-92)**	**50 (36-64)**	**97 (93-98)**
	25		**81 (65-93)**	77 (70-83)	35 (24-47)	96 (92-98)

## Discussion

The present study was carried out to review cutoffs adjusted for education in a southern Brazilian sample. A ROC Curve was built to establish the MMSE cutoffs. The cutoffs presented in the results showed the best balance of sensitivity and specificity values. To determine most of the cutoffs, we focused on the sensitivity, to keep the MMSE characteristic of a screening instrument. However, for some cutoffs we could not emphasize sensitivity because this lost specificity without increment of PPV or NPV.

The best cutoff in the total sample was 23, with sensitivity of 86% and specificity of 83%. This cutoff has been used worldwide.^17-21^ The best cutoff for the illiterate group was 21 and the best cutoffs for the lower, middle and higher educational level groups were 22, 23 and 24, respectively. The sensitivity values of these cutoffs were 93%, 87%, 86% and 81%, respectively. The specificity values of these cutoffs were 82% for the cutoffs 21 and 22, and 87% for the cutoffs 23 and 24. These MMSE cutoff points showed good diagnostic results for detecting dementia.

Most of the demographic variables differed significantly between the dementia patients and the healthy participants. However, these characteristics did not influence the establishment of the coordinates in the ROC curve analysis.

The present study showed results that corroborated the need for different cutoffs which take into account the educational level when evaluating cognitive deficits. This differentiation is important to prevent mistaken diagnosis of dementia cases.^23,26^ Misdiagnosis of healthy individuals as dementia patients may cause distress for the subject and their family, besides unnecessary expenses.

Examining previous investigations conducted in Brazil, which also considered the educational level and reported sensitivity and specificity for different cutoffs, reveals that each study used different tests for the determination of the cutoff points, and the division of the educational levels was also different. Bertolucci and coworkers^26^ used the Kolmogorov-Smirnov test to select the cutoff points. Their results were: 13 for the illiterate group (Sensitivity=82.4%, Specificity=97.5%), 18 for lower educational level group (Sensitivity=75.6%, Specificity=96.6%) and 26 for middle educational level group (Sensitivity=80%, Specificity=95.6%). Almeida^20^ used contingence 2x2 tables to determine the values of sensitivity and specificity for the cutoffs and suggested a cutoff of 19/20 for illiterate elderly people (Sensitivity=80%, Specificity=71%) and 23/24 for elderly with some level of education (Sensitivity=84%, Specificity=60%). Lourenço and Veras^34^ used, as did the present study, a ROC curve to select the cutoff points, suggesting a cutoff of 18/19 for illiterate individuals (Sensitivity=73.5%, Specificity=73.9%) and 24/25 for individuals with some educational level (Sensitivity=75%, Specificity=69.7%).

A limitation of our study was the small number of dementia patients in the sample. When the sample was subdivided by educational level, few dementia patients fell into each group, especially in the middle and higher education groups, compared to the great number of healthy elders.

Besides educational attainment, cognitive evaluation performance may be influenced by a number of other factors such as previous abilities, social and cultural contexts, language, interviewer training, and the environment in which the test is run.

It is important to note that elderly individuals' complaints on cognitive problems are not predictive of objective cognitive decline^40^ and that subjects with an objective decline may not present a cognitive complaint.^41^ Therefore, it is important that the evaluation of cognitive aspects become a part of the routine medical evaluation of elderly patients.^40,41^

As we showed in our previous study^31^, primary education in Brazil is highly heterogeneous with regional characteristics, a factor interfering in studies that evaluate cognitive performance. The sociological studies and educational evaluations have shown that the educational systems reflect social inequalities, and result in different learning outcomes for the same number of years of schooling.^42^ This characteristic ultimately limits the use of universal cutoff points and raises the importance of regional studies.
